# An Adult *Loa loa* Worm in the Upper Eyelid: An Atypical Presentation of Loiasis in the United States

**DOI:** 10.1155/2021/6630875

**Published:** 2021-04-16

**Authors:** Linnet Rodriguez, Julia Michelle White, Nikisha Q. Richards, Alan X. You, Natario L. Couser

**Affiliations:** ^1^Department of Ophthalmology, Virginia Commonwealth University Health System, 401 N 11th St, Richmond, VA 23219, USA; ^2^Departments of Internal Medicine and Emergency Medicine, Virginia Commonwealth University Health System, 417 N 11th St, Richmond, VA 23298, USA; ^3^Department of Human and Molecular Genetics, Virginia Commonwealth University Health System, 1101 E Marshall St, Richmond, VA 23298, USA; ^4^Department of Pediatrics, Virginia Commonwealth University Health System, Children's Hospital of Richmond at VCU, 1000 E Broad St, Richmond, VA 23219, USA

## Abstract

*Purpose*. To report a case of ocular involvement of Loa loa parasite. *Observations*. We present a rare case report of a Loiasis diagnosed in the United States from a patient presenting with subcutaneous migration of an adult worm within an eyelid who was found to have systemic disease with microfilaria in his blood. This is the second report in the United States and the eighth case in published literature worldwide. *Conclusions and Importance*. Due to the relatively mild disease course, Loiasis is relatively ignored in public health in low resource health districts. Understandably, the focus of public health in endemic areas must focus on basic health needs like malnutrition and diseases that entail a greater disease burden. As globalization has increased the amount of trade of physical goods, the effect of immigration also has implications for the spread of infectious disease. Medical practitioners in the United States should be aware of endemic diseases from foreign lands.

## 1. Introduction

*Loa loa* is a common filarial parasite in Western and Central Africa which infects an estimated ten million people or more [1]. Loiasis, the infection caused by this parasite, is asymptomatic in most people infected [2]. Common symptoms of the disease include Calabar swelling, or localized episodes of angioedema, and subconjunctival migration of adult worms [3]. Other ocular structures have also been reported to be affected by Loa loa infection. Therefore, the main objective of this paper is to report a case of Loa loa involvement of an eyelid as it is described below.

## 2. Case Presentation

A 28-year-old healthy male presented to the Emergency Department (ED) with an intermittent “movement sensation” within the left upper eyelid which began 12 hours prior to his presentation.

The patient reported “stinging” pain during the episodic eyelid movements. He denied previous occurrences of this “movement sensation” as well as fever, pruritus, or recent insect bites. Our patient reports possible history of “a tapeworm” in childhood but did not recall any treatment or additional details. Born in Cameroon, the patient immigrated to the United States ten years prior to his presentation. He denied any foreign travel within one year. Two years prior to presentation, he resided in several Caribbean islands including Dominica, Saint Kits, and Barbados.

**Figure 1:**
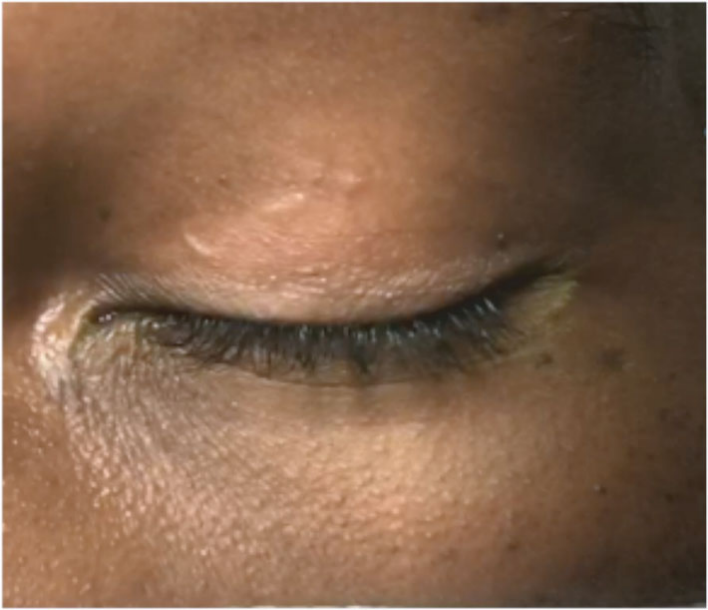
External photograph of left periorbital region which demonstrates a thin, serpentine lesion of the left upper eyelid near the eyelid crease.

External examination of the left upper eyelid revealed a subcutaneous thin cylindrical lesion (Figure 1). The patient intermittently noted a painful “movement sensation” of the left upper eyelid. At that time, medical practitioners noted visible episodic movement and slow migration of the subcutaneous lesion. No erythema, edema, or overlying break in the skin was noted. The patient noted that exposure to bright light stopped the “movement sensation” within his eyelid.

General physical exam and complete ophthalmologic exam, including dilated fundoscopy, were unremarkable.

**Figure 2:**
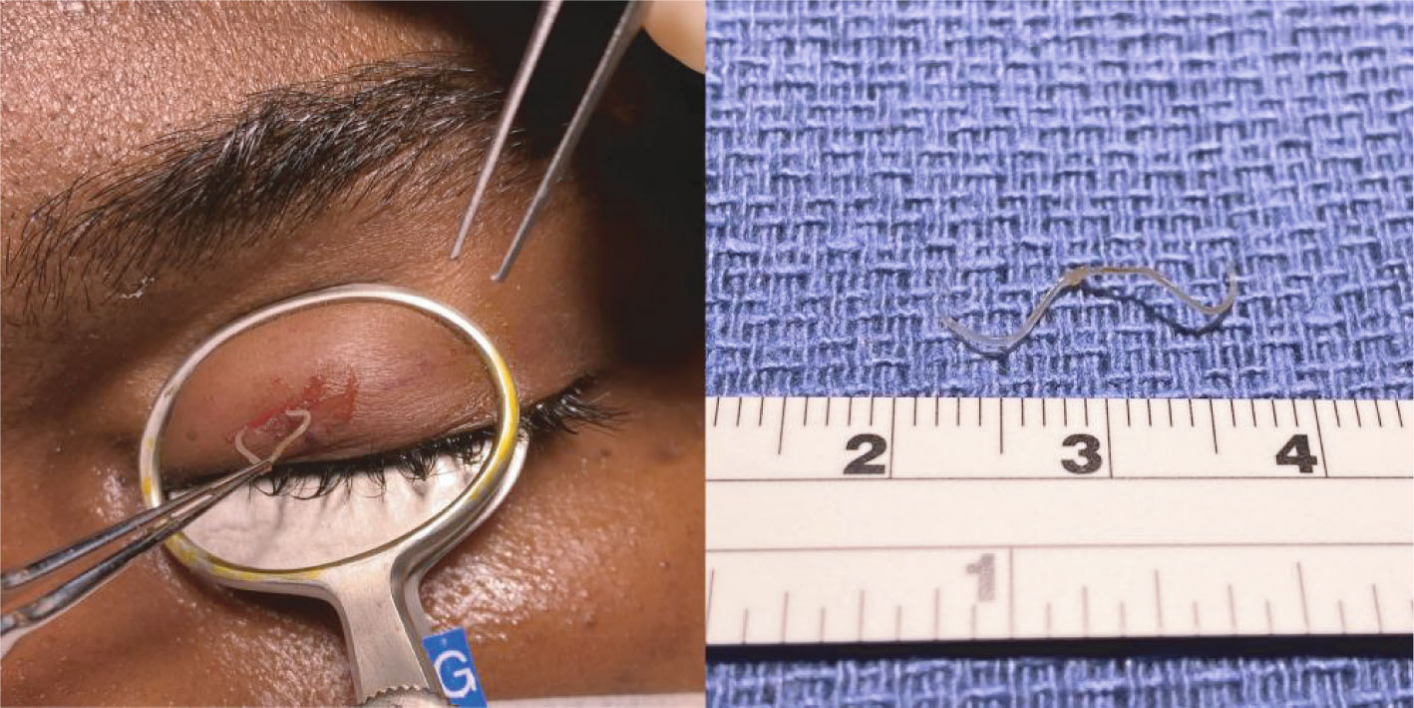
Surgical extraction of male adult Loa loa worm from the left upper eyelid and gross specimen at right which measured 3.2 cm from head to tail.

Extraction of the subcutaneous worm was performed in a sterile bedside procedure. Topical anesthesia was applied to the left globe, and local anesthetic was delivered subcutaneously in the eyelid. The location of the worm was marked with ink. A chalazion clamp was placed to aid with excision and confinement of the mobile lesion. A five-millimeter horizontal incision was made just superior to the worm. Blunt dissection was performed to identify the lesion of interest, and the slender 3 cm long white worm was removed (Figure 2, Video 1). The specimen was sent to microbiology and pathology for analysis.

Pathology examination of the gross specimen revealed an adult male *Loa loa* worm. Therefore, this is the first reported case in the United States of a male *Loa loa* worm.

Peripheral blood smear revealed microfilaria. The CBC and BMP were unremarkable except for a mildly elevated ALT of 61. Notably, there was no eosinophilia.

This patient followed with the infectious disease department at our academic medical facility. Microfilarial load of *Loa loa* is being calculated. Onchocerca serologic testing was negative. Patient begun a 21-day-course of oral diethylcarbamazine to decrease the *Loa loa* filarial load. Repeat filarial testing is planned in one year to monitor the disease.

## 3. Discussion

Loiasis is a filarial disease caused by infection with the nematode *Loa loa*. Known colloquially as African eye worm, Loiasis is transmitted to humans by the bites of tabanid flies like *Chrysops silica* and *Chrysops dimidiate*, which introduce larvae into the subcutaneous tissues of human hosts [3]. Over six to twelve months, the larvae develop within human subcutaneous tissues into adult worms, which measure 30-70 millimeters (mm) in length and 0.3-0.5 mm in diameter [3]. Once mature, the adult worms continue traveling through the subcutaneous tissue at rates up to one centimeter per minute [3]. Adult worms have been reported to survive in human tissue for up to 21 years [4]. Immature larvae or microfilariae are released by adult female worms and migrate between the host's bloodstream and lungs in a diurnal pattern [3].

Currently, it is estimated that greater than 10 million people are infected with *Loa loa* [1]. Endemic areas encompass much of Western and Central Africa with the highest prevalence of disease being in Cameroon, Gabon, Equitorial Guinea, Congo, and the Central African Republic [5]. Though exceedingly common in Western Africa, Loiasis is most often asymptomatic [3]. Symptoms from the disease may emerge years after initial infection [3]. The most common sign of Loiasis is Calabar swellings, which are episodes of localized angioedema most often on the face and extremities [2]. Calabar swellings are caused by a hypersensitivity response to parasitic antigens in subcutaneous tissues [3]. Often painless, Calabar swellings may be painful if involving the joints [3].

A second hallmark of Loiasis is subconjunctival migration of adult worms, which may be associated with conjunctivitis, epiphora, foreign body sensation, and transient eyelid swelling [3]. These symptoms are typically self-limited. While the benign subconjunctival migration of adult *Loa loa* worms is common, other ophthalmic manifestations have been reported, though rarely. Intraocular adult filaria have been noted in the anterior chamber, which may cause corneal edema, uveitis, hypopyon, and secondary cataract formation [6–10]. In patients with disseminated Loiasis and encephalopathy, retinal hemorrhage, retinal artery occlusions, vitreous hemorrhage, and chorioretinitis have been noted [11, 12]. In one case, pathological specimens of the retina showed numerous microfilaria within the retinal vasculature with the concentration of microfilaria corresponding to the degree of retinal edema and hemorrhage [11].

Other more serious but rare systemic complications of Loiasis are reported including meningoencephalitis, hematuria, proteinuria, endomyocardial fibrosis, pleural effusions, arthritis, and lymphangitis [12]. These complications are thought to be due to the inflammatory reaction to microfilarial antigens [11].

To our knowledge, this case represents the second case of periocular subcutaneous *Loa loa* macrofilaria of the eyelid in the United States. Our literature search has only revealed eight similar cases previously recorded in medical literature (the appendix). This is the first male *Loa loa* worm extracted from the periocular subcutaneous tissues. In two of the previously published cases, it was noted that exposure to a bright light source induced movement of the filaria [13, 14]. On the contrary, our patient stated that bright light caused cessation of movement of the adult worm.

Diagnosis of *loa loa* may be accomplished by microscopic detection of microfilariae in the peripheral blood. The microfilaria presence in the serum follows a diurnal curve, with a high density between 10 : 00 and 16 : 00 [3]. Blood drawn outside this window may yield a false negative result [1]. It is interesting to note that our patient was found to have microfilaria in blood drawn at 20 : 00. Unfortunately, up to half of *L loa* infected patients do not have detectable microfilariae in their blood, which makes laboratory diagnosis difficult [1]. PCR test is also available in some locations to detect *L loa* specific DNA [3]. Clinical diagnosis may be necessary, and criteria would include exposure to an endemic area, Calabar swelling, and subconjunctival macrofilaria.

Treatment of Loiasis is difficult as its medical treatment poses significant side effects. Coordination of care with an infectious disease or tropical medicine specialist is vital. The first-line treatment, diethylcarbamazine (DEC), utilizes the patient's immune response to kill both microfilaria and adult worms [3]. Complete treatment may require repeated doses. In patients with high loads of microfilaria, DEC treatment entails a significant risk of encephalopathy [1]. Though this side effect is not well understood, it is thought to be due to sudden decomposition of larvae resulting from DEC treatment [1]. Second line treatments include ivermectin and albendazole [1]. Both are limited in efficacy because they only act to kill one life stage of the parasite. Ivermectin treatment is lethal to microfilaria but not adult larvae [3]. In addition, the treatment with ivermectin may also lead to encephalopathy in patients with high microfilarial loads [1]. Albendazole is thought to kill adult parasites by inhibiting microtubule formation and the uptake of glucose, but it does not affect microfilaria [3]. In a few cases in countries with advanced medical systems, apheresis has been used to decrease the burden of microfilaria from the blood which has then allowed for treatment with DEC without adverse effect [15].

There has been no elimination campaign for *Loa loa* due to the relatively low burden of disease caused by the parasite but also due to serious adverse effects from treatment with antihelminthic drugs [16]. Due to the coendemic nature of onchocerciasis and Loiasis, patients treated for either parasitic disease must be tested for the other infection in order to prevent toxic and potentially lethal side effects from medical treatment [3]. Loiasis treatment with DEC is contraindicated in patients coinfected with Onchocerca as the treatment may worsen Onchocercal eye disease [3]. Onchocerciasis treatment with ivermectin may produce severe side effects including encephalopathy, cardiomyopathy, and nephropathy [14, 17]. Patients with a high microfilarial load of *L loa* have been identified as those at highest risk [17].

## 4. Conclusion

Loiasis is an underrecognized and undertreated infectious disease in Central and Western Africa. Though rarely diagnosed in developed nations, practitioners in all medical specialties must be aware of the disease which may present in immigrants and travelers up to 21 years after their initial infection. This case report notes a novel clinical presentation of Loiasis in the United States. A young, healthy male presented with Loiasis ten years after his most recent exposure to an endemic area, Cameroon. He had no hallmark ocular symptoms nor Calabar swelling during the previous ten years, yet he was found to have an adult male *L loa* worm in his eyelid. Microfilaria were noted in his blood sample drawn at night, which is outside the typical midday peak for microfilarial concentration in the blood. Surgical removal of the adult male filaria from the upper eyelid allowed for prompt diagnosis. This is the first case in which a patient noted light to stop filarial movement. Treatment of infection with *Loa loa* requires coordination with infectious disease specialists and can be complicated by incomplete eradication of the filaria and adverse reactions from the treatment.

## Data Availability

Please refer to the appendix and references listed in the case report.

## Appendix

### Previously Published Cases of Periocular Subcutaneous Adult *Loa loa* in Chronological Order

1. 32-year-old Zambian woman found to have an asymptomatic female *Loa loa* worm in the left lower eyelid [13]
2. 23-year-old African student living in Germany presented with “acute, recurrent, multifocal episodes of pain and swelling of his left upper eyelid” for 3 weeks and was found to have a female filaria [18]
3. 35-year-old Ghanaian male living in the United Kingdom for eight years presented with one day history of left eye redness which had intermittently occurred for the preceding 6 years. A subcutaneous worm was removed from the left upper eyelid [19]
4. 32-year-old Indian woman presented with a two-month history of “painless vermiform swelling of left upper eyelid” with intermittent “sensation of something crawling”. A female *Loa loa* worm was extracted [14]
5. 60-year-old American man with transient facial swellings had macrofilaria extracted from his left upper eyelid 21 years after travel to Nigeria for a 3-day trip [4]
6. 32-year-old male patient in Romania presented with left upper eyelid pain, and an adult worm was removed from his left upper eyelid [20]
7. 31-year-old patient from Cameroon living in France for 8 years who presented for discomfort in eyelid for several weeks found to have microfilaria on blood smear, with removal of parasite from upper pelvis subcutaneous tissue [21]

## Supplementary Materials

Supplementary Materials Video 1: video of the surgical extraction of a male *Loa loa* worm from the left upper eyelid.
